# Time course of 25(OH)D_3 _vitamin D3 as well as PTH (parathyroid hormone) during fracture healing of patients with normal and low bone mineral density (BMD)

**DOI:** 10.1186/1471-2474-14-6

**Published:** 2013-01-03

**Authors:** Christoph Wölfl, Sarah Englert, Arash A Moghaddam, Gerald Zimmermann, Gerhard Schmidt-Gayk, Bernd Höner, Aidan Hogan, Marcus Lehnhardt, Paul A Grützner, Leila Kolios

**Affiliations:** 1 Department of Trauma- and Orthopaedic Surgery, BG Trauma Centre Ludwigshafen, Ludwig- Guttmann-Str.13, Ludwigshafen 67071, Germany; 2 Department of Trauma and Orthopaedic Surgery, TKH Mannheim, Bassemannstrasse 1, Mannheim, 68156, Germany; 3 Clinical Laboratory Limbach, Im Breitspiel 15, Heidelberg, 69126, Germany; 4 Department of Social and Legal Sciences, SRH Hochschule, Heidelberg, Ludwig-Guttmann-Str.6, Heidelberg, 69123, Germany; 5 Department of Plastic-, Reconstructive and Hand surgery, Burn Care Centre, BG Unfallklinik Ludwigshafen, Ludwig-Guttmann-Str.13, Ludwigshafen, 67071, Germany

## Abstract

**Background:**

Until now the exact biochemical processes during healing of metaphyseal fractures of healthy and osteoporotic bone remain unclear. Especially the physiological time courses of 25(OH)D_3_ (Vitamin D) as well as PTH (Parathyroid Hormone) the most important modulators of calcium and bone homeostasis are not yet examined sufficiently. The purpose of this study was to focus on the time course of these parameters during fracture healing.

**Methods:**

In the presented study, we analyse the time course of 25(OH)D_3_ and PTH during fracture healing of low BMD level fractures versus normal BMD level fractures in a matched pair analysis. Between March 2007 and February 2009 30 patients older than 50 years of age who had suffered a metaphyseal fracture of the proximal humerus, the distal radius or the proximal femur were included in our study. Osteoporosis was verified by DEXA measuring. The time courses of 25(OH)D_3_ and PTH were examined over an eight week period. Friedmann test, the Wilcoxon signed rank test and the Mann-Withney U test were used as post-hoc tests. A p-value ≤ 0.05 was considered significant.

**Results:**

Serum levels of 25(OH)D_3_ showed no differences in both groups. In the first phase of fracture healing PTH levels in the low BMD level group remained below those of the normal BMD group in absolute figures. Over all no significant differences between low BMD level bone and normal BMD level bone could be detected in our study.

**Conclusions:**

The time course of 25(OH)D_3_ and PTH during fracture healing of patients with normal and low bone mineral density were examined for the first time in humans in this setting and allowing molecular biological insights into fracture healing in metaphyseal bones on a molecural level. There were no significant differences between patients with normal and low BMD levels. Hence further studies will be necessary to obtain more detailed insight into fracture healing in order to provide reliable decision criteria for therapy and the monitoring of fracture healing.

## Background

Low serum levels of 25(OH)D_3_ are suggested to be associated with reduced BMD (bone mineral density) [1,2]. Lips et al. found reduced levels of serum 25(OH)D_3_ to be common amongst women with osteoporosis [3]. The diminishing ability of the skin to synthesize 25(OH)D_3_ with advancing age or a poor diet are just are some reasons [4,5].

Individuals with vitamin D deficiency are more likely to fall and to lose muscle mass [6]. Chronic insufficiency of 25(OH)D_3_ leads to secondary hyperparathyroidism with increased bone turnover, bone loss and thus a higher fracture risk [4]. As a result, guidelines for the management of postmenopausal osteoporosis typically include recommendations for its supplementation [7,8].

During fracture repair, circulating levels of 25(OH) D_3_ increase in white leghorn chickens due to an increase in CYP24A1 activity (25-dihydroxyvitamin D_3_ 24-hydroxylase, degrades 1.25-(OH)_2_D to calcitroic acid) [9]. Serum 1.25-(OH)_2_D levels after sustaining a fracture have only been measured in a few studies with humans [10]. Significantly reduced levels were found 28 hours after sustaining the fracture.

There are only few studies, observing the time course of 25(OH)D_3_ during the fracture healing, neither in healthy nor in osteoporotic bone. Meller et al. investigated on it and amongst others also on PTH in a population of 13 young adult patients without osteoporosis and long bone fractures [11] and in a population of 41 geriatric patients with proximal femur fractures [12].

The second major mediator of bone remodelling is the parathyroid hormone (PTH). PTH regulates serum calcium levels by acting mainly on bones and kidneys. Two important functions e.g. are stimulating of renal calcium re-absorption and bone resorbtion when calcium levels are low [13]. The intermittent administration of human parathyroid hormone is to date the only clinically approved bone anabolic therapy for osteoporotic patients of both genders [14].

The 84-amino-acid polypeptide hormone is an essential regulator of calcium homeostasis [15]. PTH and PTHrP (PTH-related protein) indirectly activate osteoclasts leading to increased bone resorption [16]. The hypercalcemic effect of PTH lies, in part, in its effect on the nephron enhancing renal calcium reabsorption and, in part, in bone resorption [17].

PTH infused continuously reduces BMD, increases levels of bone resorption marker TRAP-5b and raises serum calcium levels [17,18]. An intermittent application of PTH that permits complete serum clearence between doses [19] increases 1.25-(OH)_2_D and BMD, raises levels of bone formation marker osteocalcin without producing hypocalcaemia. 1.25-(OH)_2_D complements this PTH effect and may therefore facilitate an optimal skeletal response to PTH. This indicates that a full response requires an active 1.25-(OH)_2_D synthesizing system [17].

Some clinical studies indicate that a daily subcutaneous injection of PTH stimulates the development of new bone, increases bone mass in patients with osteoporosis [20] and reduces the incidence of fractures in postmenopausal women [21].

Administered during fracture healing PTH promotes bone healing in healthy and osteoporotic rats, when it is applied during early stage of healing without having any adverse systemic effect [22].

So far there is no data on the endogenic behaviour of PTH during fracture repair of healthy or osteoporotic bones. In our study, we therefore examined the time courses of 25(OH)D_3_ and PTH during healing of metaphyseal fractures of normal and low BMD level long bones.

## Methods

Between March 2007 and February 2009 all patients over 50 years of age who had suffered a metaphyseal fracture (distal radius-, proximal humerus- and proximal femur fracture) with an obligatory indication for surgical stabilization were included in this study. Exclusion criteria were defined as: polytrauma, significant soft tissue injury, high grade open fracture, > 24h mechanical ventilation after surgery, dialysis patients, long-term therapy with immunosuppressants such as corticosteroids, collagenosis, chronic inflammatory bowel disease, haematological disorders, malignant neoplasms and metabolic bone diseases (osteomalcia, hyperparathyreodism, Paget’s disease).

In all patients X-rays of the fractured region and also of the lumbar spine were performed in an anterior-posterior and lateral view. X-rays of the lumbar spine were used to exclude morphological changes of the vertebrae leading to wrong values in DXA bone density measurements. To monitor the clinical course of fracture healing, follow-up X-rays of the fractured site were performed after 4 and 8 weeks, and also one year after osteosynthetic stabilization.

BMD was examined on the lumbar spine, the entire hip on both sides and the hip subregions using dual energy X-ray absorptiometry DXA (Lunar iDPX, GE Medical Systems Germany, Solingen, Germany) based on Encore TM Version II.X software. One year after surgery follow-up DXA measurements were performed. Using normative data for young adult white people, BMD was categorized as normal, low bone mineral density, or osteoporosis, as defined by the World Health Organization. Participants with a T-score ≤ −2.5 SD were categorized as having osteoporosis.

Laboratory analysis was performed the morning after injury in fasting state. Among typical routine laboratory values, 25(OH)D_3_ and parathyroid hormone PTH were examined. Quantitative measurement was detected using chemiluminescence immunoassay “Access Ostase assay 37300” (Beckman Coulter™, Brea, USA) and electrochemiluminescence immunoassay ECLIA® (Roche, Basel, Switzerland). The laboratory values were followed-up one, four and eight weeks after suregry. The following kits where used:

Vitamin D3/25(OH)D_3_:

cobas® 25 Hydroxyvitamin D3 03314847 190, Roche

Diagnostics GmbH, D-68298 Mannheim

PTH (Draft):

cobas® Parathyroid hormone – PTH, intact 11972103

160, Roche Diagnostics GmbH, D-68298 Mannheim

Patients with low BMD levels did not receive osteoporosis medication in this period.

The study was designed as an epidemiological case–control study. After receiving the results from DXA-measuring, patients were matched in osteoporotic and nonosteoporotic groups. Further matching-criteria were: age (+/- 5 years), sex (male, female), fracture localization (proximal humerus, distal radius, proximal femur), fracture type (according to AO-classification; A-, B- or C-type fractures) and surgical technique (plate, intramedular nail). Thus we received a collective of 15 matched pairs (30 patients in total).

Statistics were performed using the software SPSS 11.0.0 (IBM Germany, Munich, Germany) and Microsoft Excel 2003/2007, Microsoft Corp. Washington, USA. By Pretrail-Power analysis of the first three patients we calculated that 15 matched pairs would be necessary to achieve statistical significance. To show comparisons over the time within one group, we first performed Friedman’s test. As post-hoc test we then performed Wilcoxon-test. We performed Wilcoxon’s rank-sum test in addition to compare both groups at one point in time. P ≤ 0.05 was considered to be significant.

## Results

Between March 2007 and February 2009 54 patients over 50 years of age who had suffered a metaphyseal fracture of the proximal humerus, the distal radius or the proximal femur were included in our study. In total 15 matched pairs were constructed according to the presented matching criteria (Table 1) and their base line demographic data (Table 2). There were 7 pairs with fractures of the distal radius, 3 pairs with proximal humeral fractures and 6 pairs with proximal femoral fractures (including femoral neck and intertrochanteric fractures).

**Table 1:** Table of matched pairs

**Patient**	**Sex**	**Age**	**Fracture localisation**	**Fracture type (AO)**	**Osteosynthesis**	**Osteoporosis (T-Score)**	**BMI (kg/m**^**2**^**)**	**Weight (kg)**	**Height (cm)**	**Creatinin (mg/dl)**
**1**	female	57	dist. radius fracture	23A3/AO	plate	-1.3	22.4	64	169	0.77
**1**	female	59	dist. radius fracture	23A3/AO	plate	-2.7	28.3	79	167	0.72
**2**	female	60	dist. radius fracture	23A3/AO	plate	-1.0	27.7	80	170	0.68
**2**	female	61	dist. radius fracture	23A3/AO	plate	-2.6	26.4	72	165	0.9
**3**	female	57	dist. radius fracture	23A3/AO	plate	-0.4	28.1	72	177	0.75
**3**	female	57	dist. radius fracture	23A3/AO	plate	-2.5	21.3	69	180	0.83
**4**	female	63	dist. radius fracture	23A3/AO	plate	0.1	22.7	71	177	0.45
**4**	female	67	dist. radius fracture	23A3/AO	plate	-3.1	28	80	169	0.73
**5**	female	66	dist. radius fracture	23A3/AO	plate	0.9	20.8	56	164	0.87
**5**	female	68	dist. radius fracture	23A3/AO	plate	-2.5	33.3	95	169	0.90
**6**	female	65	dist. radius fracture	23A3/AO	plate	-0.7	23.7	73.5	176	0.92
**6**	female	63	dist. radius fracture	23A3/AO	plate	-3.2	20.3	60	172	0.84
**7**	female	56	dist. radius fracture	23A3/AO	plate	-0.3	25.4	65	160	0.75
**7**	female	69	dist. radius fracture	23A3/AO	plate	-3.9	35.5	82	169	0.74
**8**	female	60	prox. hum. fracture	11A3/AO	plate	-1.9	40.6	136	183	0.68
**8**	female	59	prox. hum. fracture	11A3/AO	plate	-3.7	32.5	87	155	0.72
**9**	female	68	prox. hum. fracture	11C1/AO	plate	-0.4	20.9	70	183	0.66
**9**	female	71	prox. hum. fracture	11C1/AO	plate	-2.7	23.2	65	167	0.63
**10**	female	74	prox. hum. fracture	11C2/AO	plate	-1.0	33.3	75	150	1.1
**10**	female	78	prox. hum. fracture	11C2/AO	plate	-3.0	22	62	168	0.84
**11**	male	72	fem. neck fracture	31B1/AO	screws	0.3	23.5	68	170	0.89
**11**	male	75	fem. neck fracture	31B1/AO	screws	-2.6	28.4	92	180	0.67
**12**	male	64	fem. neck fracture	31B1/AO	DHS	-0.2	25.4	65	160	0.73
**12**	male	68	fem. neck fracture	31B1/AO	DHS	-2.8	30.1	84	167	0.80
**13**	female	59	intertr. fracture	31A2/AO	DHS	-1.1	29.3	77	162	0.70
**13**	female	63	intertr. fracture	31A2/AO	DHS	-2.7	20.8	60	170	0.87
**14**	female	66	intertr. fracture	31A3/AO	PFN	0.25	23.1	63	165	0.76
**14**	female	70	intertr. fracture	31A3/AO	PFN	-2.6	27.4	65	154	0.87
**15**	male	64	intertr. fracture	31A3/AO	PFN	-0.4	23.3	65	167	0.71
**15**	male	62	intertr. fracture	31A3/AO	PFN	-3.2	29.1	90	176	0.64

Matching criteria were age (+/- 5 years), sex (male, female), fracture localization (proximal humerus, distal radius, proximal femur), fracture type (according to AO-classification; A-, B- or C-type fractures) and operation method (plate, intramedullary nail). T-Score, BMI, weight, height and creatinin.

**Table 2:** Table of demographic data of the patientents showing no significant differences in their base line demographics

	**Mean**		**Standard deviation**		**p-values (*T*-Test)**
**Parameter**	**Normal BMD**	**Low BMD**	**Normal BMD**	**Low BMD**	
age	63.40	66.0	5.37	6.11	0.226
BMI (kg/m^2^)	26.01	27.11	5.27	4.75	0.556
weight (kg)	73.37	76.13	18.39	12.17	0.631
height (cm0	168.87	168.53	9.21	7.31	0.913
creatinin (mg/dl)	0.7613	0.78	0.145	0.093	0.678

**Figure 1:**
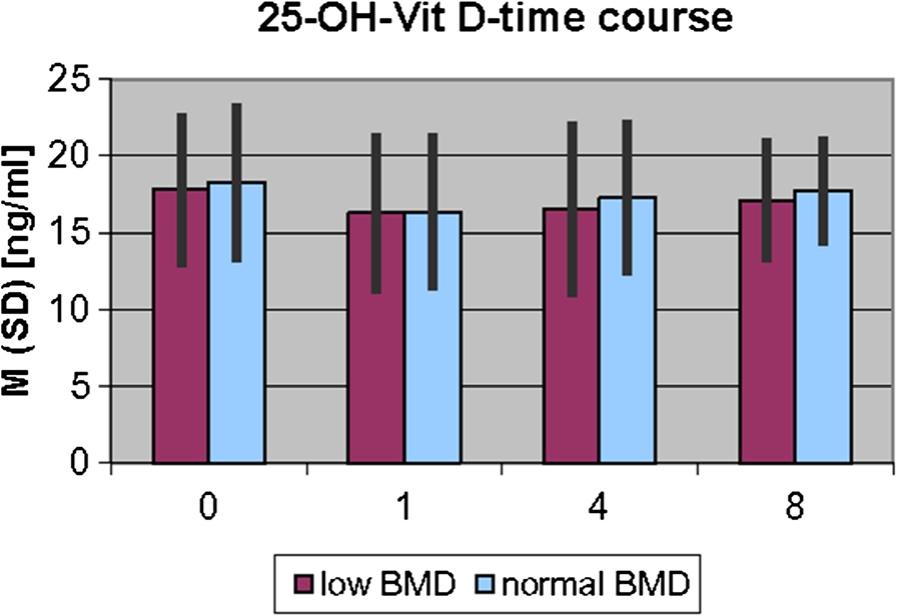
**25(OH)VitD**_**3 **_**– time course.**

**Figure 2:**
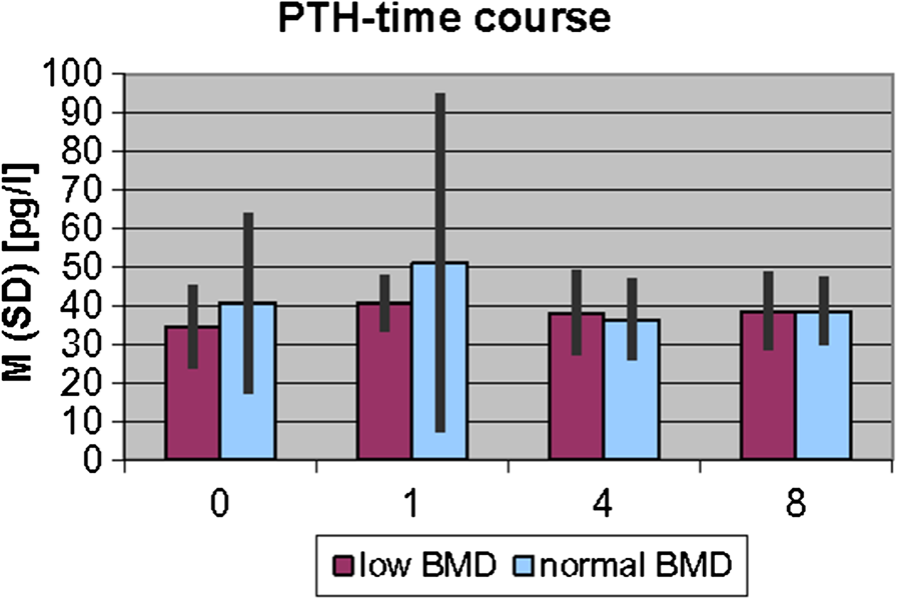
PTH time course.

The results of laboratory analyses of 25(OH)D_3_ and PTH are presented graphically in Figures 1 and 2.

Serum values of 25(OH)D_3_ during fracture healing of low BMD level patients and normal BMD level patients show no significant differences.

The time course of PTH also showed no significant changes in both groups. In the first phase of fracture healing PTH of the low BMD level group stayed below the level of the normal BMD level group.

## Discussion

Vitamin D is transformed into its active hormonal conditions through hydroxylation of hormone precursors, e.g. into 25-hydroxyvitamin D in the liver and then into 1α, 25-dihydroxyvitamin D [1.25-(OH)_2_D] in the kidneys. This active hormonal state of vitamin D plays an essential role in bone homeostasis. Upon reaching the bones as one of its target tissue, several functions are discussed. The leading effect of the vitamin D hormone on bones is indirect and mediated through its endocrine function on mineral homeostasis [23]. However, also binding its receptor (vitamin D receptor or VDR) a receptor-regulated pathway acts regulating transcription of target genes responsible for conducting the physiological process of 1.25-(OH)_2_D [24,25].

The physiological level of 25(OH)D_3_ still remains unclear and ranges from 12 ng/ml to 40 ng/ml mean [3,22]. The observed mean level of 25(OH)D_3_ in our study ranged from 16.3 ± 5.2 ng/ml to 18.3 ± 5.1 ng/ml and is similar to mean values found with other authors, describing the mean 14.7 ± 6.4 ng/ml [26].

Concerning fracture repair, the time course of 25(OH)D_3_ was examined in chicks over periods of maximal 21 days. Increasing levels of 25(OH)D_3_ were observed in the chicks due to an increase in CYP24A1 activity, whereas serum calcium levels revealed the same. Exposure of kidney cells in cultures to serum obtained from chicks with fractures showed an increase in CYP24A1 activity compared to control serum. This suggests that 25(OH)D_3_ is involved in the early stages of fracture repair and that there is some form of physiological communication between the fractured bone and the kidneys leading to an increase of renal 24-hydroxylase and the circulating concentration of this metabolite [9].

In mice CYP24a1 mRNA levels were significantly elevated in the fracture callus compared to the callus of the undamaged contralateral bone [23] and in CYP24a1-/- mice a delay in the mineralization of the cartilagous matrix of the soft callus and an altered expression of differentiation marker genes were found [23].

The presence of a nonnuclear membrane receptor for 25(OH)D_3_ in the fracture healing callus has been suggested [27,28] as well as a receptor/binding protein for 25(OH)D_3_ in the fracture healing callus membrane fraction from 25(OH)D_3_ depleted chicks [28].

Serum 25(OH)D_3_ levels after sustaining a fracture have only been measured in few studies with humans [10-12]. Significantly reduced levels were found 28 hours after sustaining the fracture.

There are two interesting studies from Meller et al. in humans in 1982 and 1984 (11,12). In the first study, regarding 25(OH)D_3_, 13 young adults who had sustained fractures of a long bone, pelvis and vertebral bodies were included. Blood samples were taken within 12 hours after sustaining the fracture and also after six to eight weeks (with the sign of primary callus). During the healing period of the fractures no significant changes could be detected in the selected two pints of time. In the second study he investigated a population of 41 geriatric patients with 43 fractures of the proximal femur. 31 fractures were treated operatively while 12 non-operatively. Again blood samples were taken at two points in time, i.e. 48 hours after administration and after 8 weeks. Concerning the time course of 25(OH)D_3_ again no significant changes could be demonstrated.

Our study is the first study to examen the time course of 25(OH)D_3_ during human fracture healing in normal BMD level bone and low BMD level bone in a time course with blood samples taken at four different points in time. We could not confirm the increase of 25(OH)D_3_ during the healing period, which we had expected, but rather a slight decrease. This heterogeneity may be explained by the different study designs, meaning results of animal studies may be different than human situation. Comparing our results with the studies of Meller, it only remains for us to show these first results as a basis for further investigations.

In addition, no significant differences could be detected between the normal and low BMD level groups in our study with regard to levels of 25(OH)D_3_. This contradicts reports of low levels of 25(OH)D_3_ in osteoporotic patients in the literature so far [1,29]. In fact, there are other authors, who failed to show any significant correlation between 25(OH)D_3_ and BMD, e.g. Garnero et al. in the OFELY (Os de Femmes de Lyon) study [30] and others [11,12,31,32] after adjusting for age.

The observed mean level of PTH ranged in our study from 34.6 ± 10.9 pg/ml to 40.8 ± 23.3 pg/ml and is similar to the mean values found of other authors, describing the mean as 47.9 ± 30.4 ng/ml [26].

Potentially, PTH can exert anabolic and catabolic effects on osteoblasts by initiating different signalling cascades [33]. To date, three receptors for PTH/PTHrP are known on osteoblasts (PTH1R-PTH3R) [34] inducing formation of cyclic 3’.5’-adenosine monophosphatase (cAMP) by activating the adenylate cyclase through stimulatory G-alpha proteins coupled to the receptor [33]. Studies in vitro and in vivo have shown that the N-terminal 1-34 synthetic fragment of the 84 amino acids of PTH mediates full PTH activity [35], eliciting a cAMP response and stimulation of bone formation [36]. Thus we propose activation of the bone anabolic effects of PTH especially in the first period after fracture, when most important stabilization processes occur.

In recent literature significant negative correlations between 25(OH)D_3_ and PTH have been described [22]. These findings support concur with the results of our study, where an increase of PTH in first week after fracture healing is accompanied by a slight decrease of 25(OH)D_3_.

One lack of our study is of course a missing control group with matching demographics without fractures, since especially 25(OH)D_3_ levels are dependent on multiple effects like oral supply or exposure to the sun. We had to accept this problem in our study design, as we rarely see patients without fractures in our trauma department.

## Conclusions

Herewith the time course of 25(OH)D_3_ and PTH during fracture healing of normal and low BMD level bone have been examined in humans in this setting for the first time providing biological insights into fracture healing in metaphyseal bones on a molecular. There were no significant differences between normal and low BMD level patients. Further studies are necessary to obtain a more detailed insight into fracture healing in order to provide reliable decision criteria for therapy and monitoring of fracture healing.

### Integrity of research and reporting

The study is approved by the local Ethical Committee of Heidelberg with the approval number 1572002. All patients gave their informed consent prior to their inclusion in the study.

## Competing interests

The authors declare that they have no competing interests.

## Authors’ contributions

CW planned the study, participated in the evaluation of patients, data, statistics and wrote the paper, he has seen and approved the final version as the corresponding author. SE participated in planning of the study, evaluation of patients and data and has seen and approved the final version. AM participated in planning of the study, evaluation of patients, data and statistics and has seen and approved the final version. GZ participated in planning of the study, evaluation of patients, data and statistics and has seen and approved the final version, HS-G participated essentially in establishment of laboratory analyses, their evaluation and statistics, but unfortunately died before submission of this study. BH participated substantially in planning of the study and performing of statistics and has seen and approved the final version. AH participated in the evaluation of patients, revision of the written manuscript and has seen and approved the final version. PAG participated in planning of the study, supervision of the study, evaluation of patients, data and statistics and has seen and approved the final version. LK participated substantially in planning of the study, supervision of the study, evaluation of patients, data and statistics and has seen and approved the final version. All authors read and approved the final manuscript.

## Pre-publication history

The pre-publication history for this paper can be accessed here:

http://www.biomedcentral.com/1471-2474/14/6/prepub
