# Correction: Time Course of 25(OH)D_3_ vitamin D3 as well as PTH (parathyroid hormone) during fracture healing of patients with normal and low bone mineral denisty (BMD)

**DOI:** 10.1186/1471-2474-15-70

**Published:** 2014-03-07

**Authors:** Christoph Wölfl, Sarah Englert, Arash Alvendi Moghaddam, Gerald Zimmermann, Heinrich Schmidt-Gayk, Bernd Höner, Aidan Hogan, Marcus Lehnhardt, Paul Alfred Grützner, Leila Kolios

**Affiliations:** 1 Department of Trauma- and Orthopaedic Surgery, BG Trauma Centre Ludwigshafen, Ludwig- Guttmann-Str.13, Ludwigshafen 67071, Germany

## 

“Time course of 25(OH)D3 vitamin D3 as well as PTH (parathyroid hormone) during fracture healing of patients with normal and low bone mineral density (BMD)” [1], the last name of Christoph Wölfl was incorrectly spelt as Wöfl. In addition, the first name of Prof. Schmidt-Gayk was incorrectly listed as Gerhard rather than Heinrich. These errors have now been corrected above.

## Pre-publication history

The pre-publication history for this paper can be accessed here:

http://www.biomedcentral.com/1471-2474/15/70/prepub
